# Forced Expression of miR-143 Represses ERK5/c-Myc and p68/p72 Signaling in Concert with miR-145 in Gut Tumors of *Apc^Min^* Mice

**DOI:** 10.1371/journal.pone.0042137

**Published:** 2012-08-02

**Authors:** Yuji Takaoka, Yuko Shimizu, Hitoki Hasegawa, Yasuo Ouchi, Shanlou Qiao, Miki Nagahara, Masatoshi Ichihara, Jiing-Dwan Lee, Koichi Adachi, Michinari Hamaguchi, Takashi Iwamoto

**Affiliations:** 1 Department of Biomedical Sciences, College of Life and Health Sciences, Chubu University, Kasugai, Aichi, Japan; 2 The Center for Education in Laboratory Animal Research, Chubu University, Kasugai, Aichi, Japan; 3 Division of Cancer Biology, Nagoya University Graduate School of Medicine, Nagoya, Aichi, Japan; 4 Department of Immunology and Microbial Science, The Scripps Research Institute, La Jolla, California, United States of America; 5 Radioisotope Research Center Medical Division, Nagoya University Graduate School of Medicine, Nagoya, Aichi, Japan; Peter MacCallum Cancer Centre, Australia

## Abstract

Recently, miR-143 and miR-145 have been shown to belong to a subset of microRNAs whose expression is controlled by a complex of a tumor suppressor p53 and DEAD-box RNA helicase subunits p68/p72. While accumulating studies have acknowledged that both miRNAs function as tumor suppressors and are similarly regulated, evidence of their coordinated action against tumorigenesis has been poorly presented. Herein, we establish transgenic mice that express miR-143 under the control of the CAG regulatory unit. When crossbred with *Apc^Min/+^* mice, the development of tumors in the small intestines is significantly attenuated. In the transgenic small intestine tumors, the endogenous miR-145 is also enhanced and the expression of c-Myc and p68/p72, both of which have been reported to be pivotal for gut tumor development, is suppressed, corresponding to the downregulation of ERK5. We demonstrate that the combination of miR-143 and miR-145 inhibits the expression of c-Myc in human colon cancer cells, whereas miR-145 retards that of p72. Moreover, we show the possibilities that miR-145 modulates p72 expression through its 3′ untranslated region and that c-Myc downregulation is involved in both p68 suppression and miR-145 induction. These findings suggest that forced expression of miR-143, probably interacting with endogenous miR-145, inhibits ERK5/c-Myc and p68/p72/β-catenin signaling and hampers small intestine tumor development in *Apc^Min/+^* mice. This unique cascade, in turn, may prevent overproduction of a subset of tumor suppressive miRNAs by repressing their own modulators, p68/p72.

## Introduction

A considerable number of lines of evidence have shown that some sets of microRNA (miRNA) act as oncogenes or as tumor suppressors, and that their biogenesis is closely linked to cancer [1], [2], [3].

miR-143 and miR-145 have emerged as tumor suppressing miRNAs, particularly for colon cancers. Michael et al. initially reported that the expression of miR-143 and miR-145 was downregulated in many colorectal neoplasms, suggesting their potential action as tumorsuppresors [4]. Indeed, this notion was supported by the following studies, which revealed that the downregulation of miR-143 and miR-145 could be involved in B-cell malignancy [5] and that colon tumor cell proliferation was suppressed by transfection with miR-143 [6].

However as most of these studies used synthetic miR-143 and miR-145 mimics, their expression was transient and usually far beyond the level normally expressed in living organisms. Hence, their physiological relevance remains to be determined.

Colorectal cancer is one of the most common cancers in not only the Western world but also Japan. Mutations in the Adenomatous polyposis coli (*APC*) gene are responsible for familial adenomatous polyposis (*FAP*) syndrome, and are usually an initial event in sporadic cancer development. *APC* encodes a large multidomain protein that binds to β-catenin and promotes its destruction to downregulate the Wnt signalling pathway [7], [8].

*Apc^Min/+^* mice, which were established by random mutagenesis with ethylnitrosourea, harbor a heterozygous truncating mutation at codon 850 of Apc and develop tens of small intestinal polyps when on the C57BL/6 background, while quite a few tumors develop in colons [9], [10].

RNA polymerase II initially transcribes long primary miRNAs (pri-miRNAs) which are generally more than several kilo base pairs (bp) and possess a 5′ cap and poly (A) tail. miRNAs mature through a multistep modification with two members of the RNaseIII endonuclease protein family, Drosha and Dicer [11]. Another RNA binding protein, DGCR8, has been found to interact with Drosha and to be a requisite for the processing of pri-miRNAs. Together with other RNA binding molecules such as DEAD-box RNA helicase subunits p68/p72 (also known as DDX5 and DDX17, respectively), Drosha and DGCR8 form a huge complex, Microprocessor [12]. Indeed, the p68/p72 knockout mice study demonstrated that p68/p72 were required for the processing of pri-miRNAs of a subset of miRNAs, such as miR-145 and miR-16 [13].

Moreover, Suzuki et al. reported that the processing of pri-miR-143 and pri-miR-145 required the interaction of the tumor suppressor p53 and the Drosha complex through the association with p68/p72 in colon cancer cells, suggesting that the full expression of miR-143 and miR-145 might be involved in tumor suppressing signaling driven by p53 [14].

On the other hand, accumulating evidence has shown a possible implication of p68/p72 for β-catenin signaling in gut tumor development. The interaction of p68/p72 and β-catenin could play a pivotal role in Wnt/β-catenin signaling, to activate the downstream effectors such as c-Myc, cyclin D1 and c-jun [15]. PDGF-induced tyrosine-phosphorylated p68 associated with β-catenin and promoted the epithelial-mesenchymal transition of human colon cancer cells, suggesting the potential implication of p68 in colon cancer metastasis [16].

Here, we establish transgenic mice in which miR-143 is ubiquitously expressed in a variety of organs. When crossbred with these mice, the development of small intestine tumors of *Apc^Min/+^* mice is retarded. Interestingly, endogenous miR-145 is also increased in these tumors. Molecular examination shows that protein expression of extracellular signal regulated kinase (ERK5), p68/p72 and c-Myc is strongly suppressed. We also present that the expression of c-Myc and p72 is downregulated by miR-143/miR-145 and miR-145, respectively, in a human colon cancer cell lines, DLD-1 and Lovo cells. The reporter assay shows that p72 could be a direct target of miR-145.

As far as we are aware, this is the first report that miR-143 suppresses tumors spontaneously developing in living organisms. This study may also provide a unique model where tumor suppressive miRNAs and the key regulators for their biogenesis, p68/p72, form a regulatory circuit.

## Results

### Forced Expression of miR-143 Induced miR-145 Expression in the Small Intestine Tumors of *Apc^Min/+^* Mice and Suppressed the Tumor Development

**Figure 1:**
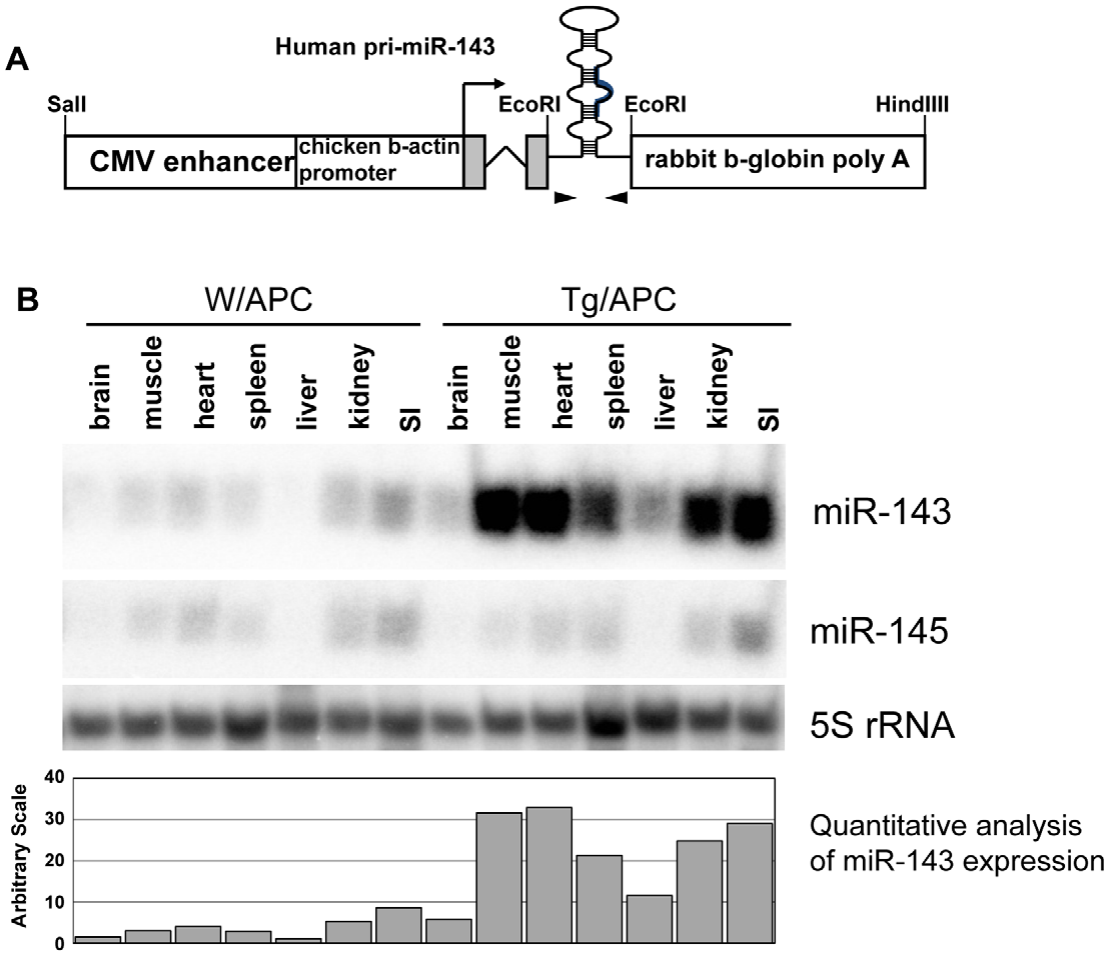
Establishment of CAG/miR-143 transgenic mice and Northern blot analysis. A) Schematic depiction of the DNA construct and its possible transcript. ∼2.6 kb SalI and HindIII fragment of CAG/miR-143 was injected to mice eggs. The shadow boxes and a bent arrow represent the 5′untranslated region and a possible transcriptional start site, respectively. Approximate positions of primers for genotyping (arrowheads) are shown. B) Polyacrylamide gel Northern blot analysis of various tissues of the transgenic mouse. Twenty µg of total RNA of various tissues from a transgenic mouse (Line C) and its normal littermate was applied in each lane. The membrane was hybridized with the probes for miR-143(1^st^ panel), miR-145 (2^nd^ panel) and 5S rRNA (3^rd^ panel). The 4^th^ panel represents quantitative analysis of miR-143 expression in the 1^st^ panel. SI: small intestine.

To express miR-143 ubiquitously in whole body, we made a construct which carried ∼300 bp human pri-miR-143 fragment under the CAG regulatory unit, composed of CMV enhancer and chicken β-actin promoter, and injected it into the fertilized mice eggs [17] (Fig.1A). We obtained four founder mice, and three of them transmitted the transgene to offspring. Since only one strain (Line C) strongly expressed miR-143, we used this strain for further analysis (Fig.1B and Fig. S1A).

**Figure 2:**
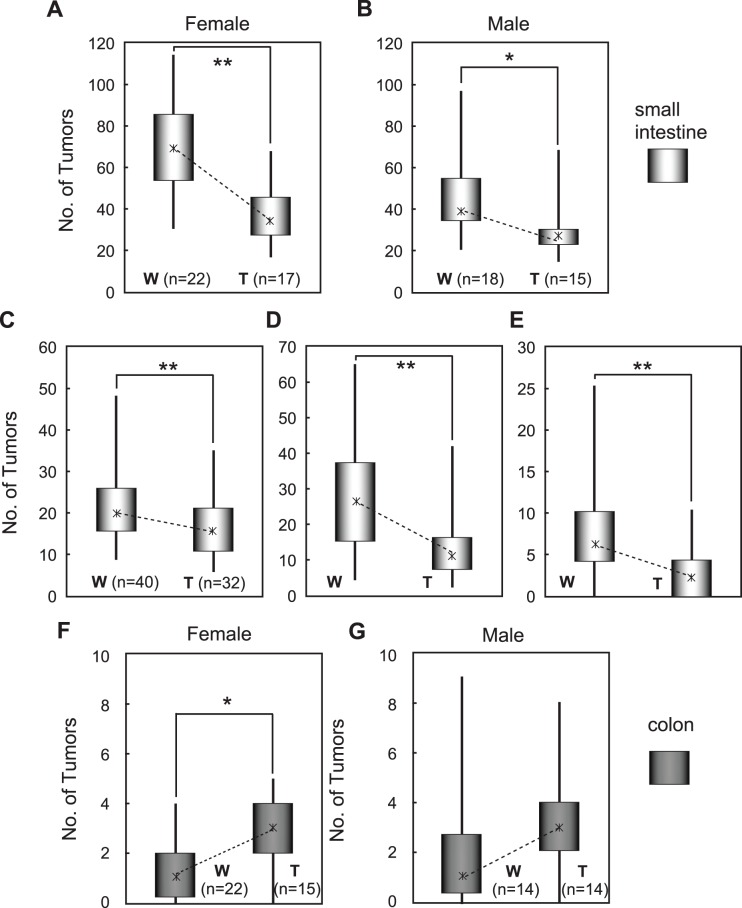
Tumor incidence in *Apc^Min/+^* mice with or without CAG/miR-143 transgene. Tumors of intestines were observed under a stereoscopic microscope. A and B) The number of small intestine tumors whose diameter (Φ) was more than 1 mm of females (A) and males (B) of W/APC (W) and Tg/APC (T) is presented. C-E) Incidence of small intestine tumors of W/APC (W) and Tg/APC (T) is presented based on tumor size: (C) 1 mm < the diameter of tumors (Φ) <2 mm, (D) 2 mm <Φ<3 mm, (E) 3 mm <Φ. F and G) The number of colon tumors whose diameter (Φ) was more than 1 mm of females (F) and males (G) of W/APC (W) and Tg/APC (T) is presented. Tumor incidence is presented by box and whisker plots and median values are given as asterisks. n: numbers of mice examined.

The transgenic mice of Line C have no abnormality in appearance. After backcrossing the mice to C57BL/6 mice four times, we crossbred the transgenic mice with *Apc^Min/+^* mice and dissected 4 month old mice to examine the tumor development (Fig. S1B). Interestingly, the small intestine tumor incidence was significantly suppressed in *Apc^Min/+^* mice carrying the transgene (hereafter referred to as Tg/APC) (Fig. 2A, 2B, 2C, 2D, 2E). On the other hand, the colon tumors developed in Tg/APC at higher frequency than non-transgenic littermates (hereafter referred to as W/APC) (Fig. 2F and 2G). As far as we examined, all the tumors of Tg/APC were adenomas and histologically showed no apparent difference from those of W/APC (Fig. S2).

**Figure 3:**
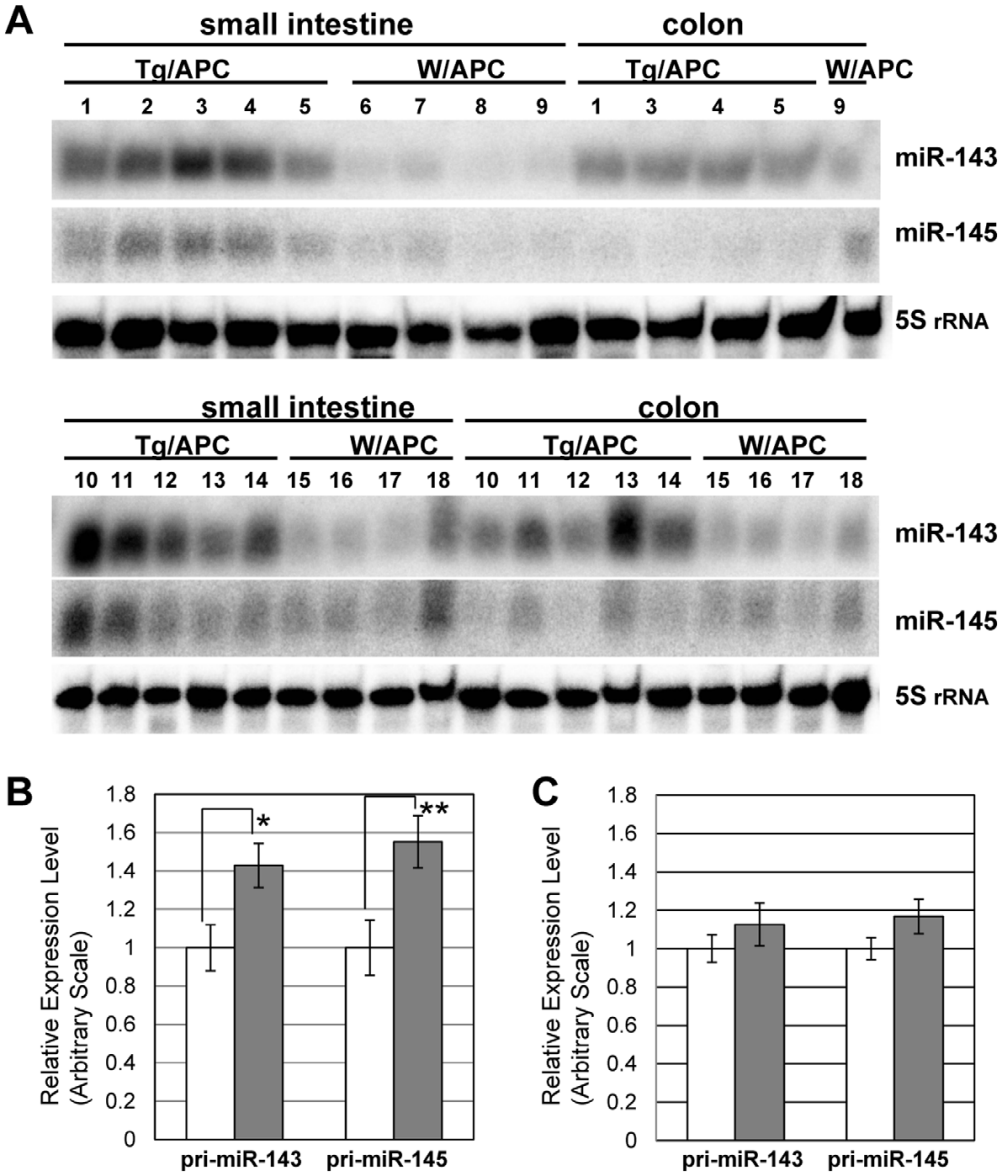
RNA analysis of gut tumors in the transgenic mice. A) Polyacrylamide Northern blot analysis of expression of miR-143 and miR-145 of gut tumors. Ten µg of total RNA of small intestines and colons of Tg/APC and their non-transgenic littermates (W/APC) was applied in each lane. The membrane was hybridized with the probes for miR-143(upper panel), miR-145 (middle panel) and 5S rRNA (lower panel). Individual identification number is presented. B) Quantitative Real-Time PCR (qRT-PCR) analysis of the mouse endogenous pri-miR-143 and pri-miR-145. The expression of mouse pri-miR-143 and pri-miR-145 of the small intestine tumors from Tg/APC (gray bars, n = 5) and their non-transgenic littermates (W/APC) (open bars, n = 4) was examined. Each set of primers cover the region that contains pre-miR-143 or pre-miR-145. The data are representative of three independent experiments and are presented as the mean ± SEM. C) qRT-PCR analysis of the mouse endogenous pri-miR-143 and pri-miR-145 of the colon tumors. The expression of mouse pri-miR-143 and pri-miR-145 of tumors from Tg/APC (gray bars, n = 3) and their non-transgenic littermates (W/APC) (open bars, n = 3) was examined in the same way as B).

As shown in Fig.3A, miR-143 was highly expressed in the small intestines tumors of Tg/APC whereas the colon tumors generally expressed lower. Thus, the sufficient expression of miR-143 appears to restrain tumor development in living animals.

Unexpectedly, the expression of miR-145 of transgenic small intestine tumors also increased in proportion to that of miR-143 (Fig. 3A). In contrast, we detected only little enhancement of miR-145 in Tg/APC colon tumors even though one of the tumors examined strongly expressed miR-143 (see Fig.3A #13). Thus, the induction of endogenous miR-145 appears to work preferentially in the small intestine polyps.

miRNAs are initially transcribed as several kilo bp pri-miRNAs, which are finally cleaved into mature miRNAs through the intermediates, ∼65 bp stem-loop precursor miRNAs (pre-miRNAs). miR-143 and miR-145 were transcribed as a bicistronic unit, a common pri-miRNA, in DGCR8-null embryo bodies [18]. To examine whether the upregulation of miR-145 in Tg/APC tumors was due to the increase of pri-miRNA, we performed qRT-PCR analysis of the mouse endogenous pri-miR-143 and pri-miR-145 with two sets of primers covering each pre-miRNA region. Figure 3B indicated that both of pri-miR-143 and pri-miR-145 expression was upregulated in the small intestine tumors of Tg/APC. Thus, the forced expression of miR-143 in the small intestine tumors may induce the transcription of a bicistronic pri-miR-143/miR-145 to promote the expression of miR-145, and possibly miR-143. Since no techniques, however, have been available to discriminate the human and the mouse mature miR-143, we could not confirm an auto-regulatory loop of miR-143. On the other hand, neither of pri-miR-143 nor pri-miR-145 significantly increased in the transgenic colon tumors (Figure 3C). Hence, the failure of miR-145 induction in transgenic colon tumors seemed to occur at the transcriptional level.

### ERK5-c-Myc Signaling was Suppressed in the Transgenic Small Intestine Tumors

Next, we tried to identify the direct target molecule of miR-143 which would contribute the retardation of small intestine tumors. So far, several candidates for miR-143 targets in cancer cells have been identified. Extracellular signal regulated kinase 5 (ERK5) has been the most intensively studied so far. Esau et al. [19] initially documented ERK5 as a target in differentiating adipocytes and other investigators confirmed this notion in some human and rodent cancer cells [5], [20].

**Figure 4:**
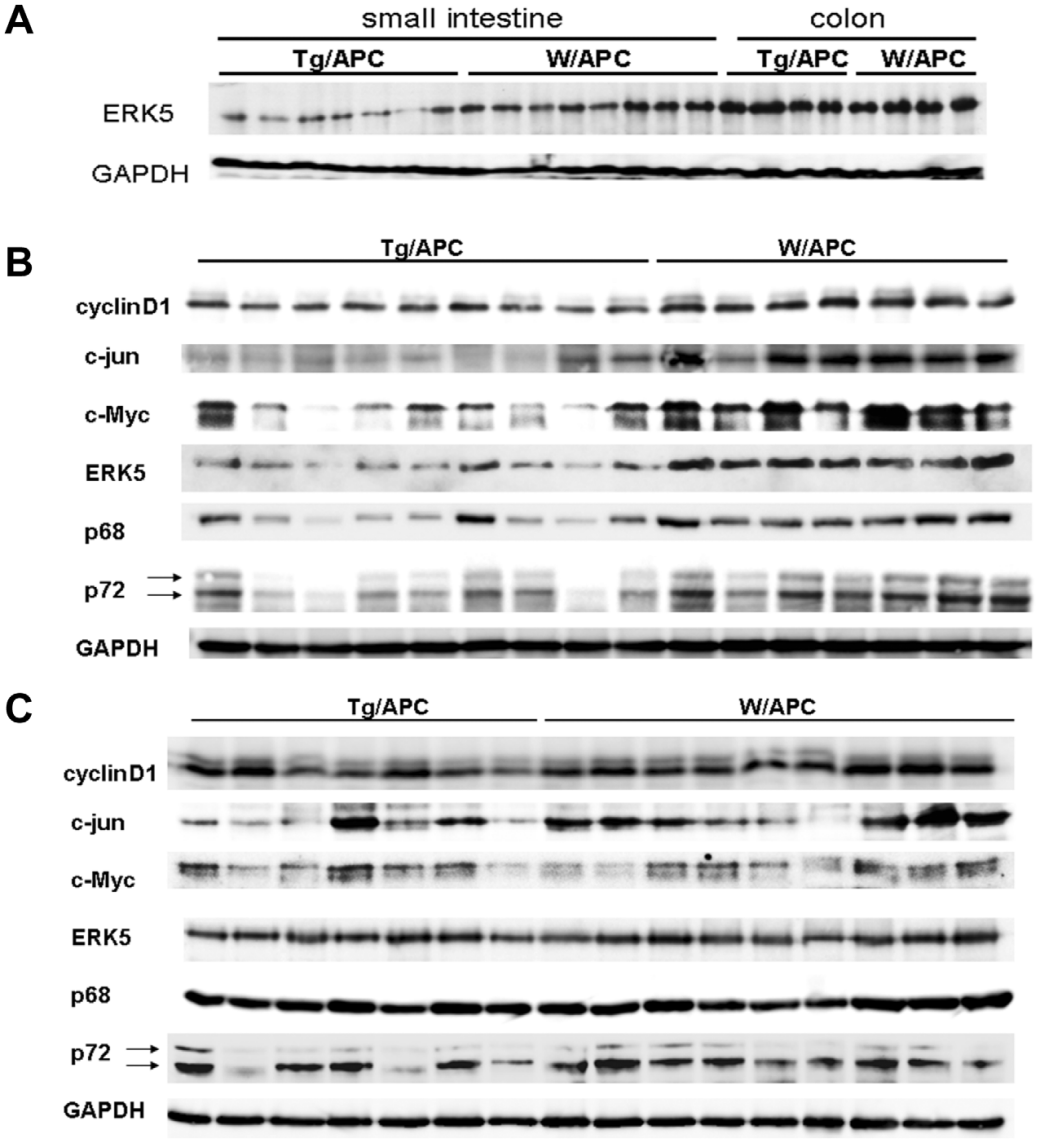
Western blot analysis of the gut tumors. A) Whole cell extracts of the small intestine and colon tumors from Tg/APC and their non-transgenic littermates (W/APC) were examined by anti-ERK5 antibody. B, C) Whole cell extracts of the small intestine tumors (B) and the colon tumors (C) from Tg/APC and their non-transgenic littermates (W/APC) were examined by the indicated antibodies. Lower and upper arrows indicate p72 and its alternatively spliced form p82, respectively.

We examined the ERK5 expression in tumors by Western blotting. Corresponding to the miR-143 expression, ERK5 expression in the small intestine tumors of Tg/APC was downregulated compared to W/APC, whereas no significant difference was observed in the colon (Fig. 4A). Thus, a significant level of miR-143 expression might be necessary for ERK suppression, or colon cells might have low sensitivity of ERK5 to miR-143. Then, we examined the expression of cyclinD1 and c-jun that have been shown to be the downstream effectors of ERK5 signaling. As shown in Fig. 4B (1^st^ and 2^nd^ panels), both expression was downregulated in the transgenic small intestine tumors.

c-Myc plays a central role in regulation of the proliferation of a variety of cells. Interestingly, the level of c-Myc was closely related to that of ERK5 (Fig. 4B 3^rd^ and 4^th^ panels), strongly implicating that c-Myc was one of the major downstream targets of miR-143 through ERK5.

**Figure 5:**
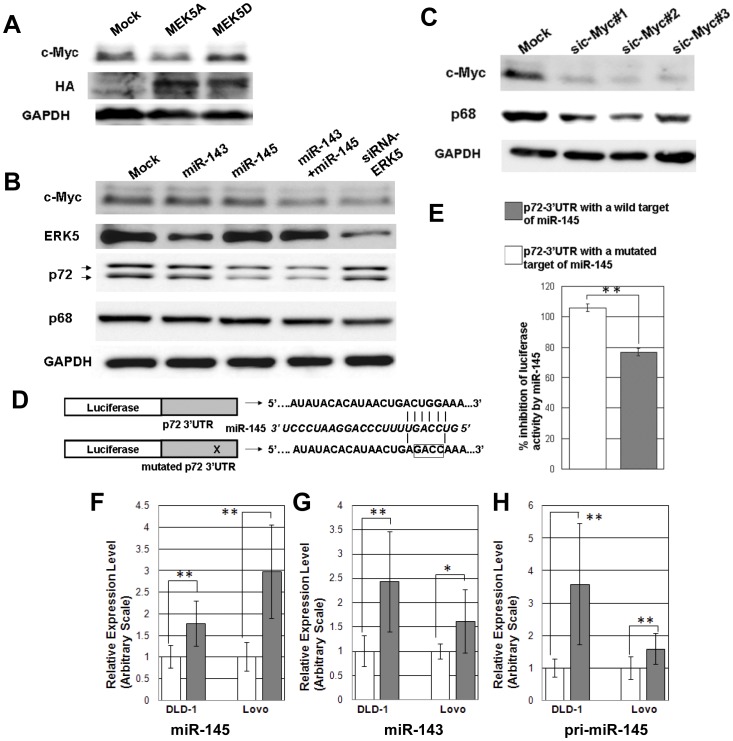
Analysis in human colon cancer cells. A-C) Western blot analysis of the transfectants. Whole cell extracts were immunoblotted with the indicated antibodies. A) DLD-1 cells in 24-well culture plates were transfected with 1 µg of dominant negative MEK5 (HA-MEK5A) or 1 µg of constitutive active MEK5 (HA-MEK5D). pcDNA3 vector was transfected as a mock. B, C) DLD-1 cells in 24-well culture plates were transfected with 20 p mol of negative control siRNA (Mock), each miRNA mimic or siRNAs for ERK5 and c-Myc. 10 p mol of each miRNA mimic was transfected for a combination of miR-143 and miR-145. sic-Myc #1∼3 represent siRNAs for c-Myc. Lower and upper arrows indicate p72 and its alternatively spliced form p82, respectively. D) Schematic depiction of pGL3-Promoter plasmids fused to the 3′UTR fragment of p72 containing a potential target site of miR-145 and its mutant. Mutated sequences are boxed. Complementary pairs of miR-145 and its target within the seed sequence are shown as vertical bars. E) % inhibition of luciferase reporter activity of the wild and mutant transfectants by miR-145 mimic is shown. F) qRT-PCR of miR-145 (F), miR-143 (G) and pri-miR-145 (H) in DLD-1 cells and Lovo cells. Cells in 24-well culture plates were transfected with 20 p mol of negative control siRNA (open bar), or siRNAs for c-Myc#1 (gray bar). The data are representative of at least two independent experiments and are presented as the mean ± SEM.

Although c-Myc was proven to be a substrate of ERK5, it was not clear whether the expression of c-Myc was regulated by ERK5 [21]. Since ERK5 is activated by MEK5, we tried to examine the effect of ERK5 activation on c-Myc expression by introducing dominant-negative MEK5A or constitute active MEK5D into human colon cancer cells DLD-1 [22]. As shown in Fig. 5A, the constitutive active MEK5D enhanced the c-Myc expression, whereas the dominant-negative MEK5A rather decreased it. Comparable results were also obtained in human embryonic kidney cells HEK293 (Fig. S3A). Hence, ERK5 activation might stabilize c-Myc protein. Intriguingly, Lee et al. recently demonstrated that activation of another member of the MAP kinase family, ERK stabilized c-Myc protein by phosphorylation in *Apc^Min/+^* mice, suggesting the potential consequence of c-Myc stabilization by the MAP kinases in their tumorigenesis [23].

Next, we examined the effect of miR-143, miR-145 or siRNA for ERK5 on c-Myc expression in DLD-1 cells. Consistent with the results of MEK5 transfection, siRNA for ERK5 clearly repressed c-Myc expression (Fig. 5B, 1^st^ and 2^nd^ panels), suggesting that ERK5 could be one of the molecules through which miR-143 regulates c-Myc.

c-Myc expression was not significantly suppressed by either miR-143 or miR-145 mimic when individually transfected. However, when expressed in combination, the effect was much pronounced. (Fig. 5B, 1^st^ panel). It should be noted that miR-143 mimic significantly downregulated the expression of ERK5 although it was weaker than the effect of siRNA for ERK5 (Fig. 5B 2^nd^ panel). Since Sachdeva et al. recently revealed that miR-145 when activated by p53 directly suppressed c-Myc expression, it is likely that miR-143 and miR-145 complement each other to downregulate c-Myc expression in our experimental systems [24]. This synergistic effect of miR-143 and miR-145 on c-Myc downregulation was also observed in another human colon cancer cell line, Lovo (Fig. S3B, 1^st^ panel).

### p68/p72/β-catenin Signaling was Suppressed in the Transgenic Small Intestine Tumors

DEAD-box RNA helicase subunits p68/p72, which are components of Microprocessor, promote the processing of pri-miR-143 and pri-miR-145 [13], [14]. Thus, we investigated their expression in transgenic tumors. Unexpectedly, both the expression p68 and p78 was drastically inhibited in the small intestine tumors of Tg/APC (Fig.4B 5^th^ and 6^th^ panels). This finding was exciting, since a recent study elegantly proved that p68/p72 were critically involved in β-catenin signaling in colon cancers [15]. Interestingly, c-Myc,cyclin D1 and c-jun are also the prominent targets of β -catenin/T-cell factor.

Hence, we examined the effect of miR-143 and miR-145 on p68/p72 expression in DLD-1 cells. As shown in Fig. 5B (3^rd^ panel), miR-145 mimic inhibited p72 expression, while neither miR-143 mimic nor siRNA for ERK5 seemed to have significant effect, which data demonstrate that the downregulation of p72 in the small intestine tumors of Tg/APC may be mainly elicited by miR-145. miR-145 also suppressed the expression of p78 in Lovo cells (Fig.S3B, 2^nd^ panel).

Analysis by TargetScan indicated that there existed one possible binding site of miR-145 in the 3′untranslated region (3′UTR) of p72 of many animal species. Thus, miR-145 might directly bind the mRNA of p72 and restrain the expression of p72. To test this idea, we constructed a fusion gene of luciferase reporter gene and the p72 3′UTR harboring one miR-145 target sequence, and subjected it to reporter assays. A construct with mutations in the seed sequence was made for a negative control (Fig. 5D). Fig. 5E demonstrated that miR-145 mimic decreased reporter activity of vector with the wild 3′UTR of p72 by 73% of that of the mutated 3′UTR. These results implied that the downregulation of p72 observed in the transgenic small intestine tumors could be at least in part a direct effect of miR-145.

As far as we examined, none of miR-143 and miR-145 mimics significantly inhibited p68 expression in DLD-1 cells and Lovo cells (Fig. 5B, 4^th^ panel, Fig. S3B, 3^rd^ panel). Heinlein et al. [25] and Rössler et al. [26] revealed that the 5′region of p68 of mouse and human had a canonical E-box, respectively. The latter group furthermore showed that the deletion of the E-box reduced the reporter activity by half. Thus, c-Myc may be one of the candidates that positively regulate p68 expression. To address this hypothesis, we introduced three distinct c-Myc siRNAs into DLD-1 cells and analyzed the expression level of p68. As shown in Fig. 5C, these three siRNAs, all of which strongly suppressed the expression of c-Myc, decreased that of p68. These data implied that failure of suppression of p68 expression by mimics of miR-143 and miR-145 in colon cancer cells might be due to insufficient c-Myc inhibition. Therefore, it is likely that the suppression of c-Myc at least in part contributes to the downregulation of p68 expression in the small intestine tumors of the transgenic mice. These findings suggest that miR-143 and miR-145 might act in concert to inhibit both ERK5/c-Myc and p68/p72/β-catenin signaling, and thereby suppress the expression of cyclin D1, c-jun and c-Myc itself.

We also analyzed the signaling in the non-tumorous segments of transgenic small intestines. As shown in Figure S4A, the expression level of miR-145, as well as normal organs (Fig. 1B), was almost comparable between the transgenic mice and their littermates. Although the expression of ERK5 in non-tumorous segments of Tg/APC small intestines was significantly decreased, retardation of cyclin D1, c-jun and c-Myc expression was subtle or much weaker compared to that in tumors (Fig. S4B, 1^st^-4^th^ panels). The expression of p68 in non-tumorous segments of Tg/APC was inhibited but more slightly than in tumors though its overall expression level seemed to roughly correspond to that of c-Myc (Fig. S4B, 5^th^ panel), and the suppression of p72 was hardly detected (Fig. S4B, 6^th^ panel). These data suggested that forced expression of miR-143 should be sufficient for ERK5 repression, but that miR-145 induction might be a crucial event for full inhibition of cyclin D1, c-jun and c-Myc through retardation of 68/p72/β-catenin signaling.

Consistent with the ERK5 expression level, c-Myc and p68/p72 were expressed in colon tumors of Tg/APC at similar levels as W/APC, although the expression of p72 in some samples of Tg/APC was weak (Fig.4C).

### Possible Involvement of c-Myc in the Elevation of miR-145 Expression

To examine whether miR-143 induces the miR-145 expression in human colon tumor cells, we introduced miR-143 mimic into DLD-1 cells and Lovo cells, and analyzed the miR-145 expression by qRT-PCR. There was, however, no obvious enhancement of miR-145 (data not shown). Indeed, although miR-143 was strongly expressed in one colon tumor of transgenic mice, the induction of miR-145 was poor (Fig.3A #13). Hence, additional events other than miR-143 expression seemed necessary for substantial induction of miR-145 in colon cancer cells. c-Myc is an essential transcription factor in development of tumors and its expression was decreased in the tumors of the intestine but not the colon in Tg/APC. Intriguingly, c-Myc has already been proven to form the feedback loop with miR-17-92 cluster in cancer cells [27].Thus we introduced siRNA for c-Myc into DLD-1 and Lovo cells. Figure 5F showed that c-Myc siRNA enhanced the expression of miR-145 to some extent in both cells. Moreover, miR143 expression also increased by c-Myc siRNA (Fig. 5G).

To examine whether the regulation of miR-145 by c-Myc occurred at the transcriptional level, we performed qRT-PCR analysis of pri-miR-145. Consistent with the transgenic intestine tumors, the expression of pri-miR-145 of c-Myc siRNA-introduced cells was increased (Fig 5H). These data implicated that suppression of c-Myc might be at least partly involved in the elevation of miR-145 and probably of miR-143 in the transgenic intestine tumors.

## Discussion

Here, we present that forced expression of miR-143, which is *in vivo* processed from pri-miRNA, induces miR-145 expression and represses the small intestine tumor formation in *Apc^Min/+^* mice.

ERK5, also known as BMK1, is one of the MAP kinases and was shown to be involved in various types of cancers including prostate and breast cancers [28]. While its role in gut cancer development was not clear, the phosphorylation of ERK5 was increased by fetal bovine serum stimulation in Caco-2 cells [29]. Recently, ERK5 was found to be a target of miR-143 in adipocytes [19].

We demonstrated the expressions of ERK5 and its downstream effectors, c-Myc, c-jun and cyclin D1, were also suppressed in the transgenic small intestine tumors. In particular, c-Myc expression seemed to be tightly linked to the ERK5 expression level.

Of interest, c-Myc has been proven to be a critical mediator of β-catenin signaling in tumorigenesis of intestines after APC loss [23], [30], [31]. The data of our transfection experiments, together with these previous studies, suggest that miR-143 probably inhibits tumor development by downregulating c-Myc expression in cooperation with miR-145 in our transgenic mice.

Downregulation of p68/p72 could also be one of the key factors that retard tumor development in the small intestine of Tg/APC. Our results suggested the downregulation of p72 by miR-145 through binding to its 3′UTR might be at least one of the molecular basis for the decrease of its expression in the transgenic small intestine tumors.

In addition, we showed that the expression of p68 was downregulated by the introduction of c-Myc siRNAs. Thus, it is possible that p68 and c-Myc might form a positive feedback loop, which suggests that miR-143 and miR-145 might inhibit p68 in part through the repression of c-Myc.

Further analysis by TargetScan also revealed the existence of possible target sites of miR-206 and miR-34a/miR-206/miR-26a in the 3′UTRof p68 and p72, respectively, in a wide range of animal species. This was intriguing, since miR-206 and miR-26a are a set of miRNAs whose processing was proven to depend on the p53/p68/p72 complex [14]. In addition, miR-34a is a well-known p53-induced gene [32], and p68 and p72 have been shown to be strong and weak co-activators of p53, respectively [33]. Crucially, all of these were miRNAs that were found to be tumor suppressors [34], [35].

Indeed, our reporter assays showed that miR-34a (Fig. S5 B and F), miR- 206 (Fig. S5 C and G), and miR-26a (Fig. S5 D and H) mimics suppressed the reporter activity of the constructs containing each target on the p72 3′UTR, whereas miR-206 mimic also decreased that of the construct containing a target on the p68 3′UTR (Fig. S5 A and E). Although further studies are required, the p53/p68/p72 complex and a set of tumor suppressor miRNAs linked with p53 seem to constitute a novel regulatory feedback circuit.

Whereas miR-145 was induced in the small intestine tumors in Tg/APC, the molecular mechanisms remain to be obscure. Our data using DLD-1 cells and Lovo cells, however, imply that the suppression of c-Myc may be at least in part involved in its induction. Hence, given qRT-PCR analysis of pri-miR-143/145 and Western blot analysis of transgenic tumors, forced-expression of miR-143 might trigger c-Myc/pri-miR-145 signal more easily in the small intestine tumors than in the colon tumors. Interestingly, this signaling axis appeared not to exert its function efficiently in non-tumorous segments of small intestines.

Other investigators recently reported that a positive feedback circuit between miR-143 and miR-145 could work through suppressing KRAS-RREB1 signaling in pancreatic cancer cells, although they did not mention the cross-regulation at the mature miRNA levels [36]. Of note, they showed, however, this circuit was observed only in KRAS mutant cells but not in KRAS wild cells. Since KRAS mutation is not a usual event in tumors of *Apc^Min/+^* mice [9], [10], the induction of miR-145 by miR-143 in our transgenic mice would be dependent on a molecular mechanism distinct from KRAS-RREB1 signaling. Nonetheless, our study with their report indicates that regulatory circuits between miR-143 and miR-145 might exert anti-tumor effect in a variety of neoplasms in living animals.

The incidence of transgenic colon tumor development, which exhibited poor miR-143 expression, unexpectedly increased compared to non-transgenic littermates. Of interest, this result supports the recent study which compared the tumor incidence in *Apc^Min/+^* mice and *APC^loxP/+^* mice crossed with *CDX2P-NLS Cre* or *Villin-Cre* transgenic mice [37]. Their data indicate that the small intestinal tumor burden may inhibit, via an unknown mechanism, the development and/or progression of colorectal tumors in the mouse. In contrast to human cases, *APC* mutations in mice usually develop far fewer tumors in the colon than the small intestine, for as yet unknown reasons. Further studies will hopefully solve the riddle which underlies gut tumors of the human and the mouse.

**Figure 6:**
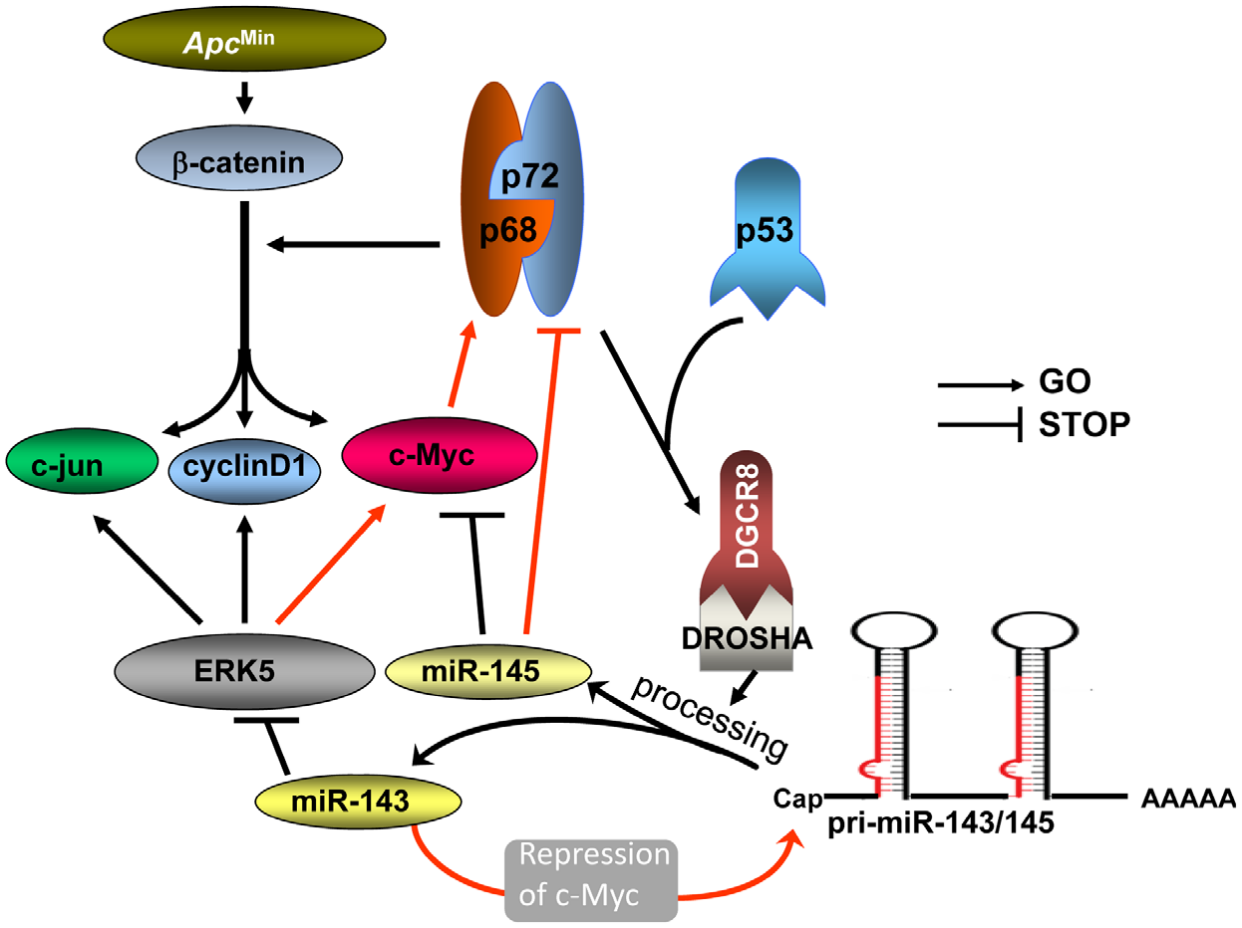
Schematic model of the regulation of APC signaling by miR-143 in the small intestine tumors. The complex of p53, p68 and p72 with DGCR8 and Drosha reinforces the processing of pri-miR-143/pri-miR-145. miR-143 downregulates the expression of c-Myc, cyclin D1 and c-jun through inhibiting ERK5 expression, and upregulates miR-145, probably by elevating the common transcript of endogenous pri-miR-143/pri-miR-145. Downregulation of c-Myc may contribute to this transcriptional augmentation of pri-miRNA. Effect of miR-143 and miR-145 converge on the repression of c-Myc, leading to the decrease of p68 expression, while miR-145 directly inhibits p72 expression. Repression of p68/p72 may impair β-catenin signaling to repress the expression of c-Myc, cyclin D1 and c-jun, and concurrently retard the processing of pri-miR-143/miR-145 to prevent the overproduction of miR-143 and miR-145. Red lines indicate the pathways which were found or confirmed in this study.

In summary, miR-143 and miR-145 likely work together to inhibit at least two signaling pathways involving ERK5/c-Myc and p68/p72/β-catenin in the intestine tumors of *Apc^Min/+^* mice, and thereby suppress their common downstream effectors. Although further studies remain to be investigated, the signaling circuit between miR-143/miR-145 and their modulators p68/p72 might also act as a guardian to arrest the overproduction of the miRNAs (Figure 6). The present study may shed new light on the network between cancer-related signaling molecules and miRNAs, which would be more diverged than expected.

## Materials and Methods

### Mice and Ethics Statement

BCF1 **(**C57BL/6N♀x BALB**/**c♂**)** females were mated with BCF1 males the night before injection and the eggs were prepared for injection as described previously [38]. Injected eggs were implanted into the oviducts of the pseudo-pregnant ICR mice. Transgenic progeny were backcrossed to C57BL/6J mice. C57BL/6J- *Apc^Min/+^*/J mice were purchased from The Jackson Laboratory. Primers for genotyping are shown in Table S1.This study was performed in strict accordance with the recommendations in Fundamental Guidelines for Proper Conduct of Animal Experiment and Related Activities in Academic Research Institutions under the jurisdiction of the Ministry of Education, Culture, Sports, Science and Technology, Japan. The protocol was approved by the Committee on the Ethics of Animal Experiments and the Institutional Animal Care and Use of Chubu University (Permit Number: 2210029). All surgery was performed under sodium pentobarbital anesthesia, and every effort was made to minimize suffering.

### Mice Intestinal Tumor Analysis

At four months of age, W/APC and Tg/APC mice were euthanized by cervical dislocation to minimize suffering and their small and large intestines were removed and flushed with phosphate buffered saline solution. Intestines were dissected and analyzed for number, location, and size of tumors with the help of a stereoscopic microscope. Some tumors were subjected to RNA and protein analysis and the others were further histologically analyzed by the Hematoxylin-Eosin staining procedure.

### Reagents

Primary antibodies for Western blot analysis are anti-ERK5 antibody:ab40809 (Abcam, Cambridge, UK), anti-c-Myc antibody: ab11917(Abcam), anti-p68 antibody: ab21696 (Abcam), anti-p72 antibody: sc-130650 (Santa Cruz Biotechnology, Inc. Santa Cruz, CA, USA), anti-cyclinD1 antibody: sc-450 (Santa Cruz Biotechnology, Inc.), anti-c-jun antibody: 2315 (Cell Signaling Technology Inc., Danvers, MA, USA), anti-GAPDH antibody: AM4300 (Ambion, Austin, TX,USA), and anti-HA antibody: 12CA5 (F. Hoffmann-La Roche Ltd., Basel, Switzerland), and second antibodies are Protein A-Horseradish Peroxidase: NA9120V (GE Healthcare UK Ltd., Buckinghamshire, UK) and Goat F(ab’)2 Anti-mouse Ig’s HRP conjugated : AMI4404 (Life Technologies Co., Carlsbad, CA, USA). siRNAs for human c-myc (#1:HS01-00222676, #2:HS01-00222677, #3:HS02-00466635) and human ERK5 (HS01-00226859), and MISSION siRNA Universal Negative Control were purchased from Sigma-Aldrich Co. (St. Louis, MO, USA). miRNA mimics of miR-143, miR-145, miR-34a and miR-26a were purchased from Qiagen GmbH (Hilden, Germany), and miR-206 mimic was obtained from Ambion.

### DNA Construction

For making the CAG/miR-143, ∼300 bp human pri-miR-143 fragment was subcloned into the EcoRI site of pCAGGS vector. To construct CAG/EGFP, the insert fragment of pMXs-puro-EGFP -miR-145/miR-143 [39] was subcloned into XhoI site of pCAGGS vector, and pri-miR-145 fragment was excised by ClaI and NotI. The ends were blunted and self-ligated. A SalI and HindIII fragment of CAG/miR-143 was purified from agarose gels with ELUTIP-D (GE Healthcare UK Ltd.), and injected into eggs. For luciferase reporter assay, the 3′UTR fragments of the mouse p68 and p72 containing possible target sites for miR-145, miR-26a, miR-34a or miR-206 were amplified from genomic DNA of a C57BL/6 mouse by PCR using specific primers containing an XhoI site at the 5′end. Each fragment was subcloned into XhoI site of the firefly luciferase pGL3-Promoter vector (Promega, Madison, WI, USA), and clones harboring inserts in the forward direction were selected by DNA sequencing. Primers for construction are shown in Table S1. No new sequence data have not been generated in this study. All experiments were authorized by the Institutional Recombinant DNA Experiment Committee of Chubu University (approval no. 06–8, 08–07).

### Cell Culture and DNA/RNA Transfection

DLD-1 cells and Lovo cells were grown in RPMI-1640 medium (#189-02025, Wako, Osaka, Japan) and Ham’s F-12 medium (#087–08335, Wako) with 10% fetal calf serum, respectively. HEK293 cells were cultured in DMEM medium (#D5796, Sigma) with 10% fetal calf serum. Transfection was performed according to the manufacture’s methods. Briefly, DNA plasmids, siRNA or miRNA was mixed with Lipofectamine 2000 (#11668, Invitrogen, Carlsbad, CA, USA) in Opti-MEMI Reduced Serum Medium (#31985, Gibco, Billings, MT, USA), then transfected into 30–40% confluent cells. Seventy two hours after the transfection, cells were subjected to analysis. DLD-1 cells and Lovo cells were purchased from Japan Health Science Research Resources Bank (HSRRB) and grown according to the HSRRB protocol and HEK293 cells were purchased from ATCC.

### Western Blot Analysis

Whole cell extracts were prepared by lysing cells in RIPA buffer (10 mM Tris-HCl, pH7.4, 150 mM NaCl, 5 mM EDTA, 1% Triton-X, 0.1% sodium dodecyl sulfate (SDS), 1% sodium deoxycholate) containing 1 mM phenylmethylsulfonyl fluoride. Fifty∼one hundred fifty µg protein was electrophoresed on SDS-polyacrylamide gels, transferred to PVDF membrane (Immobilon, Millipore Corporation, Billerica, MA, USA). The membrane was blocked by 5% skim milk in phosphate buffered saline solution with 0.05% Tween 20 (Sigma) for 30 minutes, stained with each antibody according to the manufactures’ methods and thereby subjected to ECL systems. The membrane was analyzed with Image Analyzer LAS-3000 (Fuji Film, Tokyo, Japan).

### Northern Blot Analysis

Total RNA was extracted with Trizol (Invitrogen) following the manufacture’s manual and separated on 8 M Urea 15% polyacrylamide gels for microRNA analysis, then transferred to Hybond N^+^(GE Healthcare UK Ltd.). DNA oligonucleotide probes (Table S1) complementary to each miRNA were labeled with [γ^32^P] ATP (MP biomedicals, Solon, OH, USA) by T4 nucleotide kinase (New England Biolabs, Inc., Ipswich, MA, USA). The membrane was hybridized with the probe at 42°C for 2 hours and washed 3 times with 0.2 x SSC containing 0.1% SDS at 42°C for 10 minutes. All probes were purified by MicroSpin G-25 Columns (GE Healthcare UK Ltd.) and hybridization was performed in Rapid-Hyb Buffer (GE Healthcare UK Ltd.). Data were analyzed by Typhoon Trio (GE Healthcare UK Ltd.). Densitometric analysis was performed by Multi Gauge 3.0 software (Fuji film).

### Reverse Transcription and Quantitative Real-time PCR **(**qRT-PCR)

100 n g of total RNA was subjected to the reverse transcription reaction using TaqMan Reverse Transcription Kit (Life Technologies Co.) and Taqman PCR was performed using Taqman Universal Master Mix II, No AmpErase UNG (Life Technologies Co.) on the Applied Biosystems 7300 Real-Time PCR System (Applied Biosystems, Foster City, CA, USA). The expression of mature miR-143 and miR-145 was assayed with the Taqman MicroRNA Assays (Applied Biosystems) specific for hsa-miR-143 (P/N: 4395360) and hsa-miR-145 (P/N: 4395389), respectively. snoRNA202 (Life Technologies Co., P/N: 4380914) was used for normalization. For analysis of primary transcript of pri-miRNA, total RNA was extracted by RNAqueous-R-4PCR (Ambion P/N AM1914) and subjected to qRT-PCR using the standard Taqman protocol. β-actin was used for normalization. Evaluation of β-actin expression was performed by the SYBER Green PCR. Briefly, one µg of total RNA was subjected to the reverse transcription reaction using PrimeScript RT reagent Kit (Takara Bio Co., Shiga, Japan). Then, qRT-PCR was done using SYBR premix Ex Taq II (Takara Bio Co.). All values were corrected by each calibration curve and the relative expression level was measured using the ΔΔCt method. Primers for PCR are shown in Table S1.

### Luciferase Reporter Assay

0.5µg of each construct was transfected into 30∼40% confluent DLD-1 cells that were cultured in 48-well plates w/wo 10 p mole of miRNA mimics. Seventy two hours after transfection, cells were subjected to Dual–Luciferase Reporter Assay (Promega). For normalization, a renilla luciferase vector, pRL-TK (Promega), was co-transfected.

### Statistics

Significant difference from the control was analyzed by the Mann–Whitney U test *: *p*<0.05, **: *p*<0.01.

## Supporting Information

Figure S1 **Genotyping of mice by PCR.** A) PCR analysis of genomic DNA of the transgenic mice DNA extracted from mice tail was analyzed by PCR primers for the vector and human pri-miR-143(see Fig.1A). Ethidium bromide staining image of polyacrylamide gel is shown. The arrow indicates the transgenic allele. W: wild mouse, Tg: transgenic mouse B) PCR analysis of genomic DNA of *APC^Min/+^* mice. DNA extracted from mice tail was analyzed by PCR primers for *APC^Min/+^*. The upper and lower arrows indicate the wild-type and the *APC^Min^* alleles, respectively. Ethidium bromide staining image of polyacrylamide gel is shown.
(TIF)

Figure S2 **Histological analysis of the small intestine tumors in transgenic mice.** Tumors of the intestine in four month-old mice were stained with hematoxylin and eosin. A representative small intestine tumor of a non-transgenic W/APC mouse is shown at (A) 40 × and (C) 200 × magnification. A representative small intestine tumor of its littermate Tg/APC mouse is shown at (B) 40 × and (D) 200 × magnification. Adenomatous polyps at similar differential stages developed in both mice. Black boxes indicated the areas shown in higher magnification. (bars in A and B, 1 mm; bars in C and D, 100 µm)
(TIF)

Figure S3 **Western blot analysis in cultured cells.** Whole cell extracts were immunoblotted with the indicated antibodies. A) HEK293 cells in 24-well culture plates were transfected with 1µg of dominant negative MEK5 (HA-MEK5A) or 1µg of constitutive active MEK5 (HA-MEK5D). pcDNA3 vector was transfected as a mock. B) Lovo cells in 24-well culture plates were transfected with 20 p mol of negative control siRNA (Mock) or each miRNA mimic.10 p mol of each miRNA mimic was transfected for a combination of miR-143 and miR-145. Lower and upper arrows indicate p72 and its alternatively spliced form p82, respectively.
(TIF)

Figure S4 **Analysis of non-tumorous segments of transgenic small intestines.** A)Polyacrylamide Northern blot analysis of non-tumorous segments of small intestines. Ten µg of total RNA of non-tumorous segments of small intestines of Tg/APC and their non-transgenic littermates (W/APC) was applied in each lane. The membrane was hybridized with the probes for miR-143(upper panel), miR-145 (middle panel) and 5S rRNA (lower panel). B) Western blot analysis of non-tumorous segments of small intestines. Whole cell extracts of the small intestine from W/APC and Tg/APC were examined by the indicated antibodies. Lower and upper arrows indicate p72 and its alternatively spliced form p82, respectively.
(TIF)

Figure S5 **Analysis of effect of miRNA mimics on the 3′UTR of p68 and p72 by luciferase reporter assay.** A-D) Schematic depiction of pGL3-Promoter plasmids fused to two tandem repeats of 3′UTR fragments containing a potential target site for miR-206 of p68 (A), miR-34a of p72 (B), miR-206 of p72 (C), or miR-26a of p72 (D) and corresponding mutants. Mutated sequences are boxed. Complementary pairs of each miRNA and its target within the seed sequence are shown as vertical bars. E-H) % inhibition of luciferase reporter activity of the mutant (open bars) and wild (gray bars) transfectants by miRNA mimics is shown. DLD-1 cells in 48-well plates were transfected with 0.5 µg of each pGL3-Promoter plasmid with/without 10 p mole of corresponding miRNA mimic. MISSION siRNA Universal Negative Control was used for a negative control. The data are representative of three independent experiments and are presented as the mean ± SEM.
(TIF)

Table S1 **The oligonucleotides sequences used in this study.** The oligonucleotides sequences used for genotyping, qRT-PCR and Northern hybridization are shown.
(DOCX)
