# Supplementary figures and images for: Forced Expression of miR-143 Represses ERK5/c-Myc and p68/p72 Signaling in Concert with miR-145 in Gut Tumors of ApcMin Mice
Source: PLoS One. 2012 Aug 2;7(8):e42137. doi: 10.1371/journal.pone.0042137 (PMC3410903; doi:10.1371/journal.pone.0042137)

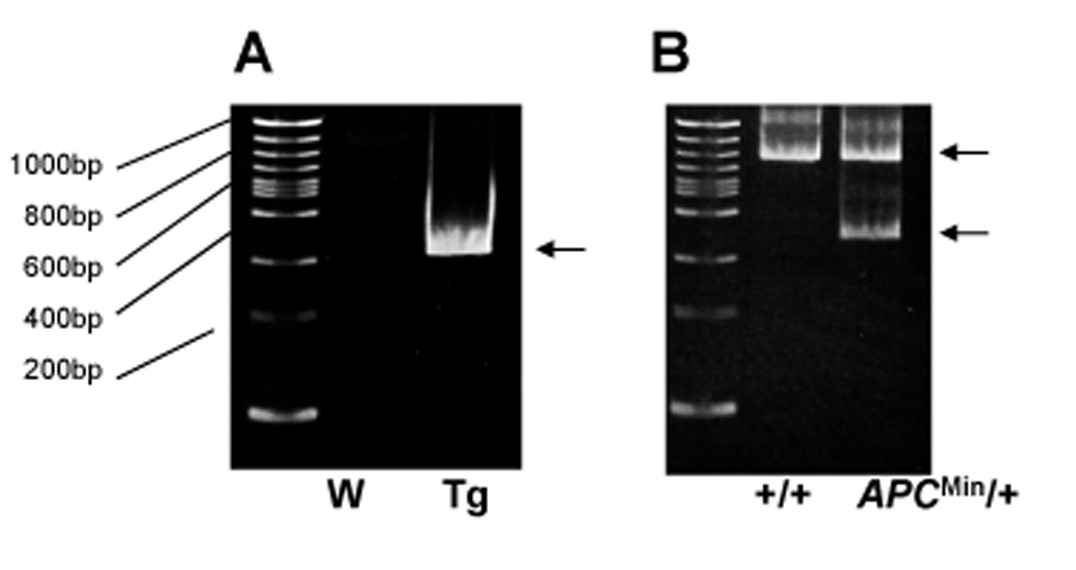

Supplement: Figure S1 — Genotyping of mice by PCR. A) PCR analysis of genomic DNA of the transgenic mice DNA extracted from mice tail was analyzed by PCR primers for the vector and human pri-miR-143(see Fig.1A). Ethidium bromide staining image of polyacrylamide gel is shown. The arrow indicates the transgenic allele. W: wild mouse, Tg: transgenic mouse B) PCR analysis of genomic DNA of APCMin/+ mice. DNA extracted from mice tail was analyzed by PCR primers for APCMin/+. The upper and lower arrows indicate the wild-type and the APCMin alleles, respectively. Ethidium bromide staining image of polyacrylamide gel is shown. (TIF) [file pone.0042137.s001.tif]

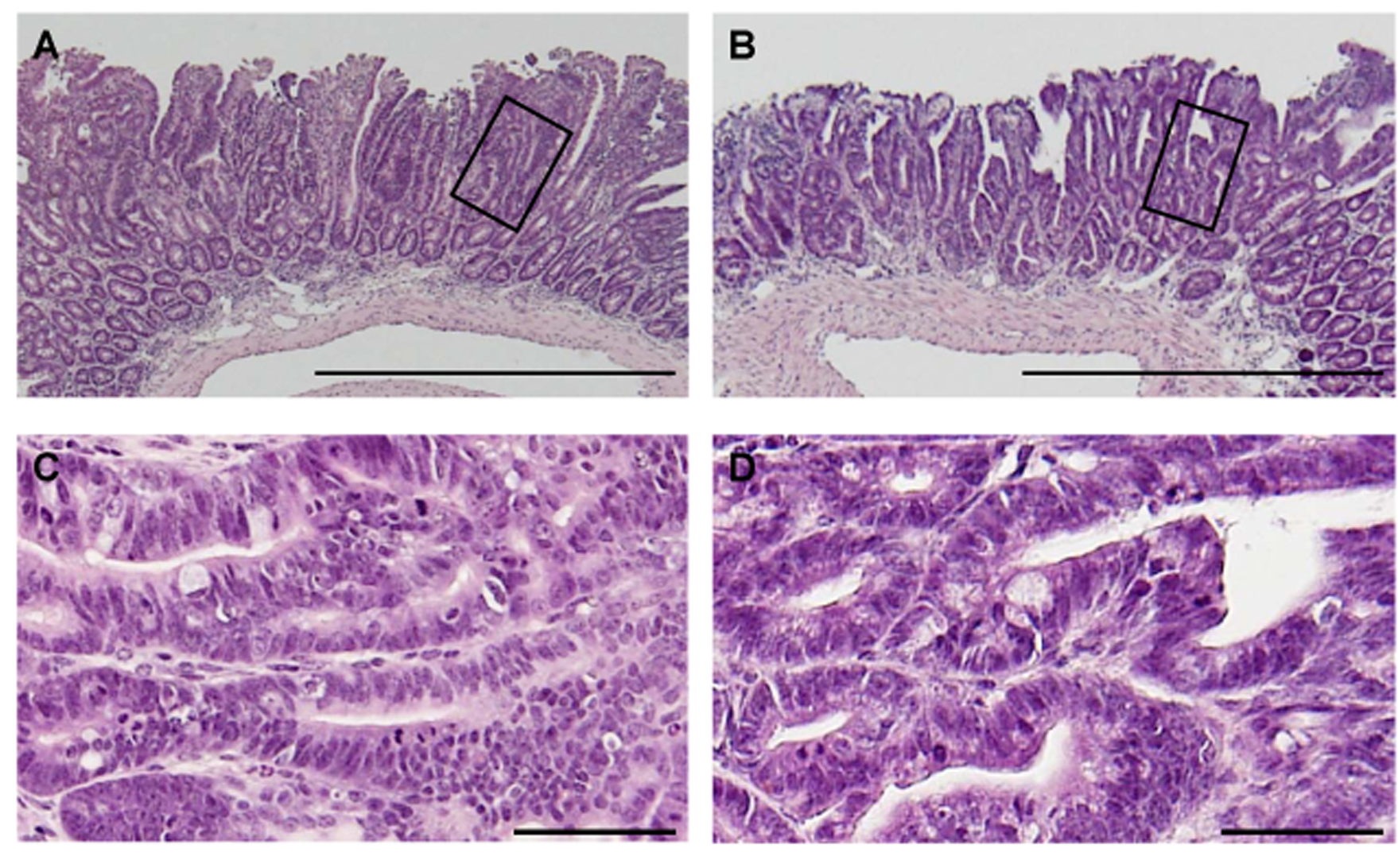

Supplement: Figure S2 — Histological analysis of the small intestine tumors in transgenic mice. Tumors of the intestine in four month-old mice were stained with hematoxylin and eosin. A representative small intestine tumor of a non-transgenic W/APC mouse is shown at (A) 40 × and (C) 200 × magnification. A representative small intestine tumor of its littermate Tg/APC mouse is shown at (B) 40 × and (D) 200 × magnification. Adenomatous polyps at similar differential stages developed in both mice. Black boxes indicated the areas shown in higher magnification. (bars in A and B, 1 mm; bars in C and D, 100 µm) (TIF) [file pone.0042137.s002.tif]

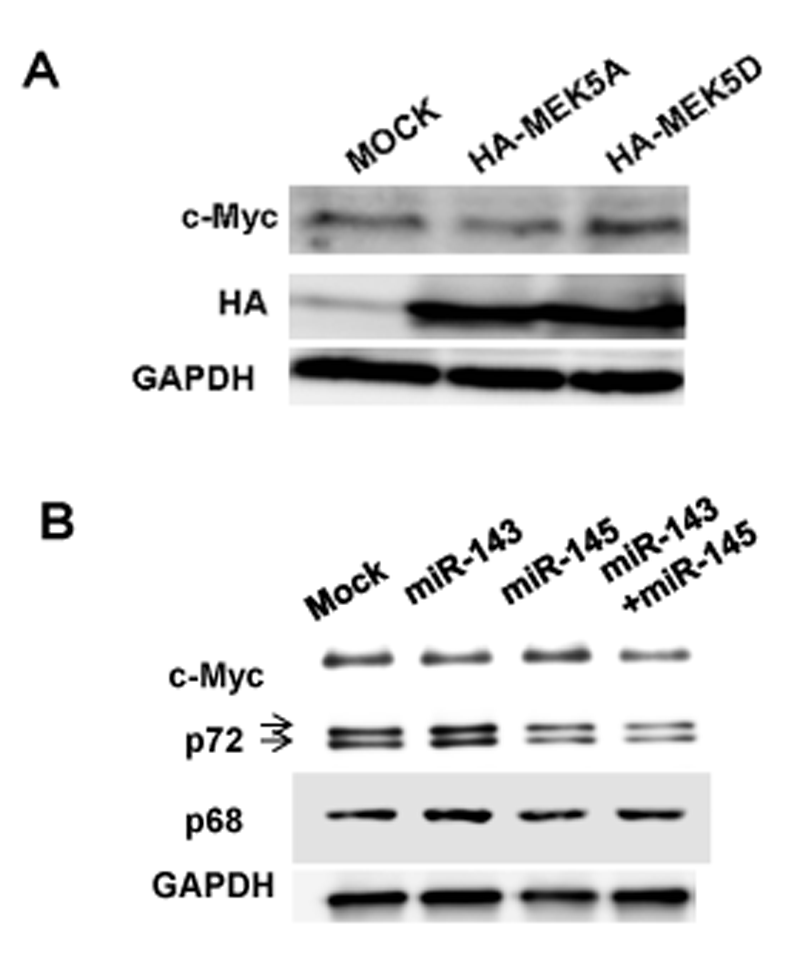

Supplement: Figure S3 — Western blot analysis in cultured cells. Whole cell extracts were immunoblotted with the indicated antibodies. A) HEK293 cells in 24-well culture plates were transfected with 1µg of dominant negative MEK5 (HA-MEK5A) or 1µg of constitutive active MEK5 (HA-MEK5D). pcDNA3 vector was transfected as a mock. B) Lovo cells in 24-well culture plates were transfected with 20 p mol of negative control siRNA (Mock) or each miRNA mimic.10 p mol of each miRNA mimic was transfected for a combination of miR-143 and miR-145. Lower and upper arrows indicate p72 and its alternatively spliced form p82, respectively. (TIF) [file pone.0042137.s003.tif]

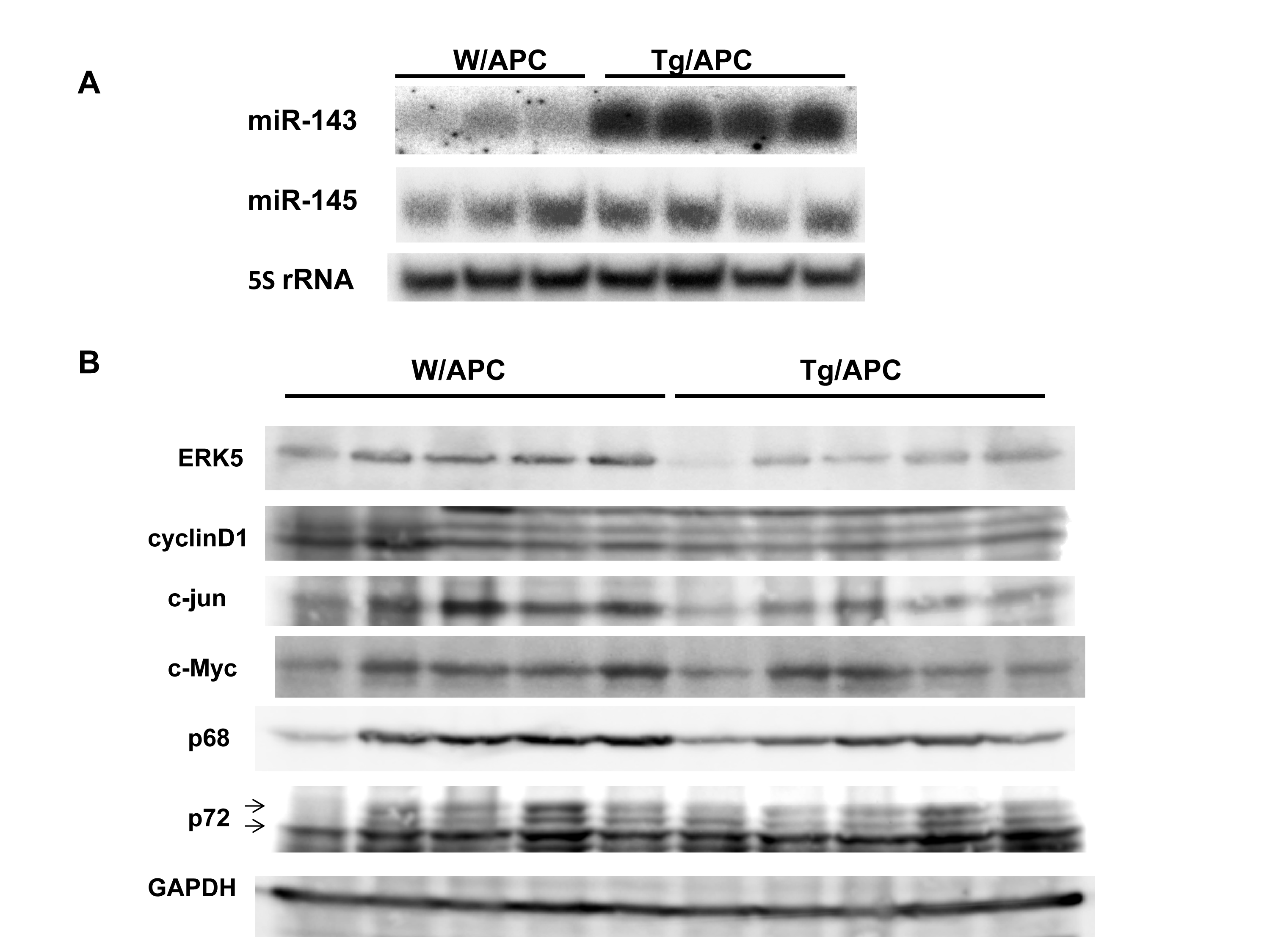

Supplement: Figure S4 — Analysis of non-tumorous segments of transgenic small intestines. A)Polyacrylamide Northern blot analysis of non-tumorous segments of small intestines. Ten µg of total RNA of non-tumorous segments of small intestines of Tg/APC and their non-transgenic littermates (W/APC) was applied in each lane. The membrane was hybridized with the probes for miR-143(upper panel), miR-145 (middle panel) and 5S rRNA (lower panel). B) Western blot analysis of non-tumorous segments of small intestines. Whole cell extracts of the small intestine from W/APC and Tg/APC were examined by the indicated antibodies. Lower and upper arrows indicate p72 and its alternatively spliced form p82, respectively. (TIF) [file pone.0042137.s004.tif]

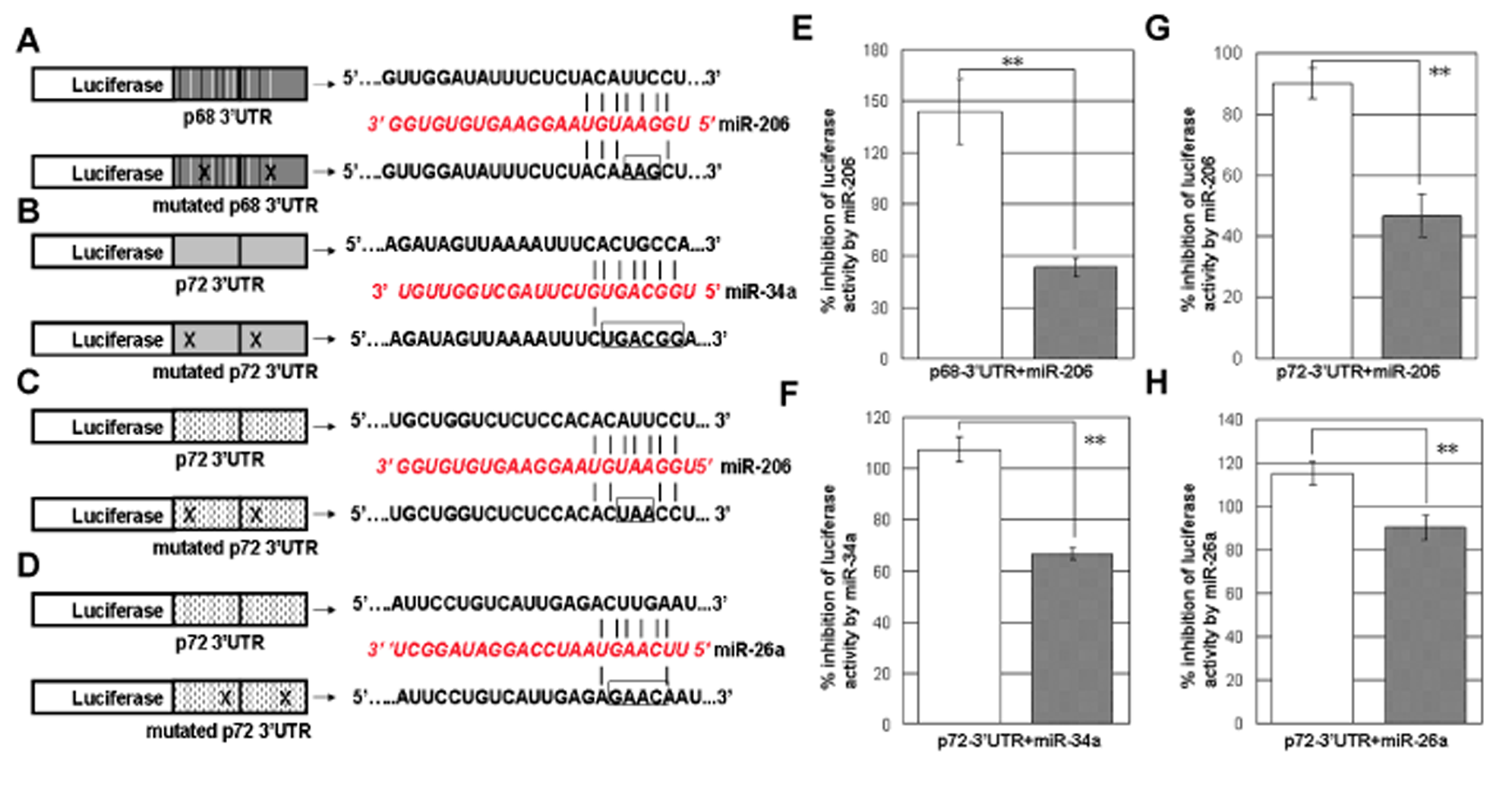

Supplement: Figure S5 — Analysis of effect of miRNA mimics on the 3′UTR of p68 and p72 by luciferase reporter assay. A-D) Schematic depiction of pGL3-Promoter plasmids fused to two tandem repeats of 3′UTR fragments containing a potential target site for miR-206 of p68 (A), miR-34a of p72 (B), miR-206 of p72 (C), or miR-26a of p72 (D) and corresponding mutants. Mutated sequences are boxed. Complementary pairs of each miRNA and its target within the seed sequence are shown as vertical bars. E-H) % inhibition of luciferase reporter activity of the mutant (open bars) and wild (gray bars) transfectants by miRNA mimics is shown. DLD-1 cells in 48-well plates were transfected with 0.5 µg of each pGL3-Promoter plasmid with/without 10 p mole of corresponding miRNA mimic. MISSION siRNA Universal Negative Control was used for a negative control. The data are representative of three independent experiments and are presented as the mean ± SEM. (TIF) [file pone.0042137.s005.tif]
