# Supplementary material for: Forced Expression of miR-143 Represses ERK5/c-Myc and p68/p72 Signaling in Concert with miR-145 in Gut Tumors of ApcMin Mice
Source: PLoS One. 2012 Aug 2;7(8):e42137. doi: 10.1371/journal.pone.0042137 (PMC3410903; doi:10.1371/journal.pone.0042137)
Supplement: Table S1 — The oligonucleotides sequences used in this study. The oligonucleotides sequences used for genotyping, qRT-PCR and Northern hybridization are shown. (DOCX) [file pone.0042137.s006.docx]

**Table S1. The oligonucleotides sequences used in this study**

| Primer | Sequence |
| --- | --- |
| CAG/miR-143 forward for genotyping | 5'-TGAGATGAAGCACTGTAGCTC-3′ |
| CAG/miR-143 reverse for genotyping | 5'-CCAGAAGTCAGATGCTCAAG-3′ |
| the wild *APC* allele for genotyping | 5'-GCCATCCCTTCACGTTAG-3′ |
| the *APC* *Min* allele for genotyping | 5'-TTCTGAGAAAGACAGAAGTTA-3′ |
| the common *APC* allele for genotyping | 5'-TTCCACTTTGGCATAAGGC-3′ |
| human pri-miR-143 forward for qRT-PCR | 5'-TCCCCTCTAACACCCCTTCTC-3′ |
| mouse pri-miR-143 forward for qRT-PCR | 5'-CACACTCCTCCTGCCCAAGA-3′ |
| human/mouse pri-miR-143 reverse for qRT-PCR | 5'-TGAGCTACAGTGCTTCATCTCAGA-3′ |
| human/mouse pri-miR-143 Taqman probe | 5'-FAM-TCTCCCAGCCTGAGGTGCAGTGCT-TAMRA -3′ |
| mouse pri-miR-145 forward for qRT-PCR | 5'-GGGATTCTGGAAATACTGTTCTTGA-3′ |
| mouse pri-miR-145 reverse for qRT-PCR | 5'-CTCCCCGGAAGGAAATGC-3′ |
| mouse pri-miR-145 Taqman probe | 5'-FAM-TCATGGCTTAGCAGCTGGATCTGTCTCC-TAMRA -3′ |
| human pri-miR-145 forward for qRT-PCR | 5’-CTCGGCTGGCTCACAGGA -3’ |
| human pri-miR-145 reverse for qRT-PCR | 5’-GCAACGCAAAGGTTTGGAACA -3’ |
| human pri-miR-145 Taqman probe | 5'-FAM-AGCCCTCTTACCTCCAGGGACAGCCT-TAMRA -3′ |
| mouse β-actin forward for qRT-PCR | 5'-CAGCTTCTTTGCAGCTCCTT-3′ |
| mouse β-actin reverse for qRT-PCR | 5'-CACGATGGAGGGGAATACAG-3′ |
| human β-actin forward for qRT-PCR | 5'-TCACCCACACTGTGCCCATCTACGA-3' |
| human β-actin reverse for qRT-PCR | 5'-CAGCGGAACCGCTCATTGCCAATGG-3' |
| mouse p68-3'UTR for Reporter assay for miR-206 forward | 5'-TCTAGAGACTGCAGTGCAGCAGTAATTATGG-3′ |
| mouse p68-3'UTR for Reporter assay for miR-206 reverse | 5'-TCTAGAACCTAAAAATTGTTTCAGGAATGTA-3′ |
| mouse p72-3'UTR for Reporter assay for miR-34a and miR-145 forward | 5'-TCTAGATTTTAATGCAGATAGTTAAAATTTC-3′ |
| mouse p72-3'UTR for Reporter assay for miR-34a and miR-145 reverse | 5'-TCTAGAAAGCTGGGGGAAAGAAATTAATTC-3′ |
| mouse p72-3'UTR for Reporter assay for miR-26a and miR-206 forward | 5'-TCTAGACTGCTAACGCCCTTGCTGGT-3′ |
| mouse p72-3'UTR for Reporter assay for miR-26a and miR-206 reverse | 5'-TCTAGAATAACATCACACCTACAATT-3′ |
| probe for miR-143　Northern hybridization | 5'-GAGCTACAGTGCTTCATCTCA-3' |
| probe for miR-145 Northern hybridization | 5’-AGGGATTCCTGGGAAAACTGGAC-3’ |
| probe for 5SrRNA Northern hybridization | 5'-TAACCAGGCCCGACCCTGCT-3' |
